# Photoperiodic plasticity of pigment-dispersing factor immunoreactive fibers projecting toward prothoracicotropic hormone neurons in flesh fly *Sarcophaga similis* larvae

**DOI:** 10.1007/s00359-024-01729-y

**Published:** 2025-01-15

**Authors:** Yutaro Ohe, Masaharu Hasebe, Yoshitaka Hamanaka, Shin G. Goto, Sakiko Shiga

**Affiliations:** 1 Graduate School of Science, The University of Osaka, 1-1 Machikaneyama-cho, Toyonaka, Osaka 560-0043 Japan; 2 Graduate School of Science, Osaka Metropolitan University, 3-3-138 Sugimoto-cho, Sumiyoshi, Osaka, Osaka 558-8585 Japan; 3 Present Address: Graduate School of Arts and Sciences, The University of Tokyo, 3-8-1 Komaba, Meguro, Tokyo 153-8902 Japan

**Keywords:** PDF, PTTH, s-LNv, Varicosity, Glutamate, sNPF

## Abstract

Larvae of the flesh fly, *Sarcophaga similis* exhibit photoperiodic responses to control pupal diapause. Although the external coincidence model is applicable to *S. similis* photoperiodism, it remains unknown how the circadian clock system integrates day-length information. To explore the mechanisms, we examined the neural circuitry involving circadian clock lateral neurons (LNs) and prothoracicotropic hormone (PTTH) neurons. We also examined the photoperiodic effects on LN-fiber patterns in third-instar *S. similis* larvae. Immunohistochemistry showed that the clock protein PERIOD and the neuropeptide pigment-dispersing factor (PDF) were co-localized in four cells per brain hemisphere, and we named these PDF-LNs of *S. similis*. Single-cell polymerase chain reaction of backfilled neurons from the ring gland showed that two pairs of pars lateralis neurons with contralateral axons (PL-c neurons) to the ring gland expressed *ptth*. Double labeling with immunohistochemistry and backfills revealed that PDF-immunoreactive varicose fibers projected close to fibers from PL-c neurons. *short neuropeptide f (snpf) receptor* and *glutamate-gated chloride channel* but not *pdf receptor* were expressed in PL-c neurons. sNPF and L-glutamate but not PDF acutely inhibited the spontaneous firing activity of PL-c neurons. The number of PDF-immunoreactive varicosities of PDF-LNs in the dorsal protocerebrum was significantly higher under short-day than that under long-day conditions in a time-dependent manner. These results suggest that sNPF and/or glutamate signaling to PTTH neurons and PDF-LNs form a potential neural circuity for the photoperiodic control of pupal diapause and that photoperiod modifies the connectivity strength between PDF-LNs and their post- or pre-neurons in the circuitry*.*

## Introduction

For seasonal adaptation, a large number of insects inhabiting temperate zones alter their physiological states for development or diapause according to photoperiod. The mechanisms underlying insect photoperiodic responses include photoreceptors, photoperiodic clocks, photoperiodic counters, and endocrine systems (Saunders 2002; Shiga 2023; Hamanaka et al. 2023). The photoperiodic clock measures the day or night length using a time measurement system, and the photoperiodic counter counts the number of photoperiodic cycles to accumulate short- or long-day information. The endogenous circadian clock system is considered to be involved in the photoperiodic clock and counter (Saunders 1982), and it has been shown that the expression of different clock genes, such as *period* (*per*) and *timeless* is a prerequisite for photoperiodism in many species (a recent review, Goto 2022, 2023). However, little is known about which clock gene-expressing cells (clock cells) are responsible (Shiga and Numata 2009), and how the circadian clock system involving brain clock cells integrates day-length and day-number information.

To address these neurobiological questions, insects that exhibit clear photoperiodic responses and possess a simple brain are advantageous. One such insect is the flesh fly larva. Flesh flies of the genus *Sarcophaga*, such as *Sarcophaga crassipalpis*, are ovoviviparous and enter pupal diapause, where development is arrested at an early pupal stage, by sensing short days (SD) at the embryonic or larval stages (Denlinger 1971). In contrast, pupae promptly develop into adults without undergoing diapause under long days (LD). Furthermore, only a five-day exposure to different photoperiods during the larval stage is sufficient to switch between diapause and non-diapause destiny (Denlinger 1971). During this short sensitive period, differences must have occurred in the brain between the SD and LD groups. In the larval brain of the fruit fly *Drosophila melanogaster* only 18 clock cells are present, whereas approximately 240 clock cells are found in the adult brain (Kaneko et al. 1997; Helfrich-Förster 2003; Reinhard et al. 2024). In the larval brains of *S. crassipalpis* and *Sarcophaga similis*, small numbers of PER-immunoreactive (-ir) cells, similar to *D. melanogaster* larvae, have been reported (Kostál et al. 2009; Yamamoto et al. 2017).

Another advantage of using flesh fly larvae is that an external coincidence model is applicable to explain the time-measurement mechanism in the photoperiodic response controlling pupal diapause in *Sarcophaga argyrostoma* and *Sarcophaga similis* (Saunders 1979; Goto and Numata 2009). In this model, LD and SD could be discriminated based on the presence or absence of light exposure during a specific phase of the circadian clock, called photoinducible phase (φi). The φi is a time window within the scotophase of the light-entrained circadian clock (Pittendrigh and Minis 1964). In both *S. argyrostoma* and *S*. *similis,* a φi of about 2 h window has been identified in the late scotophase (Saunders 1979; Goto and Numata 2009). This supports the idea that photoperiodic time measurements for the control of pupal diapause use the circadian clock system in both species (Saunders 1979; Goto and Numata 2009).

With regard to the photoperiodic response that controls pupal diapause, ecdysteroids are key players in the endocrine system. It has been shown in many lepidopteran species that ecdysteroid titers are almost shut down in the hemolymph of diapausing pupae, in contrast to non-diapause pupae (Denlinger et al. 1997; Bowen et al. 1984; Mizoguchi et al. 2013). Also in *S. argyrostoma,* ecdysteroid titers increase during adult development in non-diapause-destined pupae, whereas they are low in diapause-destined pupae (Richard et al. 1987). Interestingly, a small reduction in ecdysteroid titers appeared prior to pupation in diapause-destined larvae, suggesting that the diapause program had already manifested during the final larval instar stage in *S. argyrostoma* and the cabbage moth *Mamestra brassicae* (Richard et al. 1987; Mizoguchi et al. 2013).

In lepidopterans, ecdysteroid synthesis in the prothoracic gland (PG) is promoted by prothoracicotropic hormone (PTTH), which is produced by two pairs of neurosecretory neurons and released from the corpus cardiacum and corpus allatum into the hemolymph. PTTH titers increase prior to the increment of ecdysteroid titers (O’Brien et al. 1988; Kawakami et al. 1990; Mizoguchi et al. 2013). In *M. brassicae,* the hemolymph PTTH titer in non-diapause pupae was maintained at high levels after pupation, whereas the titer in diapause pupae decreased to undetectable levels (Mizoguchi et al. 2013). The injection of PTTH into diapause-destined pupae immediately after pupation induces adult development, showing that a lack or severe reduction of PTTH is a necessary and sufficient condition for the induction of pupal diapause (Mizoguchi et al. 2013). In flies, PTTH neurons have only been identified in *D. melanogaster* (McBrayer et al. 2007). Ablation of the PTTH neurons or *ptth*-null mutant have shown redundant control of ecdysteroid production by PTTH. This is further supported by other recent reports (McBrayer et al. 2007, Shimada-Niwa et al. 2014, Imura et al. 2020, Guirado et al. 2023). Although PTTH or PTTH neurons are not prerequisites for ecdysis, they are still important for ecdysis timing for development (McBrayer et al. 2007; Shimell et al. 2018). Therefore, it is important to examine PTTH neurons in fresh flies to understand the photoperiodic control of pupal diapause.

Interestingly, pigment-dispersing factor (PDF)-producing neurons project fibers in close proximity to the dendritic arbors of PTTH neurons in *D. melanogaster* larvae (McBrayer et al. 2007). PDF is a neuropeptide released from clock cells classified as ventral lateral neurons (LNvs) in *D. melanogaster* (Helfrich-Förster 1995; Kaneko et al. 1997). PDF functions as an output signal from the LNvs, controlling circadian activity rhythms in adults (Renn et al. 1999). PDF-ir small LNvs (s-LNvs) in the blow fly *Protophormia terraenovae* and their morphological counterpart neurons in the bean bug *Riptortus pedestris* are suggested to be involved in photoperiodic responses (Shiga and Numata 2009; Ikeno et al. 2014). *pdf* knockdown or knockout affects the photoperiodic responses in *D. melanogaster*, the brown-winged green bug *Plautia stali,* and the linden bug *Pyrrhocoris apterus* (Nagy et al. 2019; Hasebe et al. 2022; Kotwica-Rolinska et al. 2022; Kaniewska et al. 2023). Therefore, *pdf-*expressing neurons and PTTH neurons are potent candidates composing neural circuitries for photoperiodic control of pupal diapause.

In larvae of *S. similis*, PER-ir LNs and dorsal neurons (DNs) have been shown (Yamamoto et al. 2017). PER immunoreactivity changes in a circadian manner, and the changing pattern differs between LD and SD only in LNs. This suggests that PER-ir LNs may be involved in time measurement as clock cells (Yamamoto et al. 2017). In *S. similis* larvae, four pairs of PDF-ir neurons have been identified (Hirata and Shiga 2023), but it remains unknown whether PER and PDF are co-localized in the *S. similis* brain. Furthermore, PTTH neurons have not been identified and their anatomical relationship to the LNs is also unclear in *S. similis*.

In adults of *D. melanogaster,* PDF and PER are colocalized in four s-LNvs that develop from larval LNs (Kaneko et al. 1997), and their terminal fibers exhibit morphological plasticity in a circadian manner (Fernández et al. 2008, 2020). Considering the fiber plasticity of s-LNs in *D. melanogaster*, photoperiod may also cause morphological changes in the LNs of *S. similis*. The accumulation of SD or LD information may be reflected in the complexity or abundance of the fibers. The effects of photoperiod and temperature as seasonal cues have been examined in terms of the net intensity of PDF immunoreactivity in the s-LNv dorsal termini of *D. melanogaster* adults, which exhibit very shallow photoperiodism*.* The intensity of PDF immunoreactivity decreases under winter-like low temperatures (Hidalgo et al. 2023). In the case of photoperiodic-responsive species, such as *Sarcophaga* larvae, photoperiodic plasticity may occur in the LNs.

In this study, we identified PDF and PER-ir clock neurons, and PTTH neurons in *S. similis* larvae using immunohistochemistry, backfills, and single-cell polymerase chain reaction (PCR)*.* We then examined the morphological connections between the PDF-ir and PER-ir cells (PDF-LNs) and *ptth*-expressing neurons by double labeling with immunohistochemistry and backfills, and further examined transmitters received by PTTH neurons using electrophysiology. Finally, we examined the morphological plasticity of PDF-LN terminal fibers between SD and LD conditions to discuss how the circadian clock cell-PTTH neuron axis may integrate day-length information in the photoperiodic response.

## Materials and methods

### Insects

Stock cultures of *S. similis* originating from adults captured at the Toyonaka campus of the University of Osaka Japan (34.80° N, 135.45° E) were used. All experiments and breeding were performed at 20 ± 1.0 °C. Newly emerged adult males and females under LD (16 h light: 8 h dark) were transferred to SD (12 h light: 12 h dark) conditions on the day of adult eclosion and provisioned with water, sugar, and a piece of chicken liver. White-fluorescent bulb (4.4–7.7 Wm^−2^, FL15W; NEC Lighting, Tokyo, Japan) or white LED bulb (5.3–7.5 Wm^−2^, LT-N300N-YS; OHM ELECTRIC, Tokyo, Japan) were used for the light period. Females larviposited 14 d after liver feeding. Deposited larvae were set on a piece of chicken liver under LD or SD conditions one day after larviposition (Day1). Most larvae ceased feeding 5 d after larviposition (Day5) at the third-instar stage and left their food.

The diapause status of the pupae was determined 10 d after puparium formation. After removal of the puparium head capsule, diapause and non-diapause pupae were distinguished according to compound eye color. When a red color was detected in the eye region, the pupae were determined to be non-diapause. Those without a red color were determined to be diapause pupae (Fraenkel and Hsiao 1968).

For the hematoxylin-eosin staining, double labeling of PDF and PER immunohistochemistry, double labeling of PDF immunohistochemistry and backfills, and electrophysiology larvae deposited by females reared under LD conditions at 20 °C were used on Day5 under LD conditions.

### Hematoxylin–Eosin staining

The anterior larval body, including the central nervous system (CNS; brain and thoraco-abdominal ganglia), was fixed for 24 h with an aqueous Bouin fixative at room temperature (RT: 23–28 °C). After three rinses with 70% ethanol at RT, the body was dehydrated using an ethanol series and embedded in paraffin. Paraffin blocks were cut into 8-µm sections. After deparaffinization, sections were incubated in Mayer’s hematoxylin solution (Sakura Finetek Japan, Tokyo, Japan) for 5 min at RT. After washing with tap water and DW, the sections were incubated in eosin solution (Sakura Finetek, Japan) for 3 min at RT. After washing with DW, the sections were dehydrated using an ethanol series and mounted in xylene.

### Immunohistochemistry

For double labeling with PDF and PER antisera, the CNS was removed at Zeitgeber Time (ZT) 0–2 (ZT0: onset of photophase). The CNS was fixed for overnight (about 10 h) in 4% paraformaldehyde (PFA) at 4 °C. After washing with 0.1 M phosphate-buffered saline containing 0.5% Triton X-100 (PBST, pH 7.4), the CNS was incubated with 5% normal donkey serum (NDS; IHR-8135, ImmunoBioScience, Washington, USA) in PBST at RT for 3 h to block non-specific antisera binding. The CNS was incubated with goat anti-PER antiserum (1: 100, sc-398462, Santa Cruz Biotechnology, Texas, USA) and rabbit anti-PDF antiserum (1: 5,000, RRID:AB_2916037, a gift from Dr. Tomioka) as the primary antisera in 5% NDS in PBST at 4 °C for 8 d. After washing with PBST for 3 d, the CNS was incubated with 5% NDS for 1 h at RT followed by incubation with biotinylated donkey anti-goat IgG (1: 200) antiserum (705–067-003, Jackson ImmunoResearch Laboratories, Pennsylvania, USA) at 4 °C for 4 d. After washing with PBST, the CNS was processed for the amplification of PER signal using avidin–biotin complex (PK-4000, Vector Lab., California, USA) for 3 d at 4 °C. Then the CNS was incubated with streptavidin Alexa fluor 647 (1:200, S21374, Thermo Fisher Scientific, Fisher Scientific, Waltham, MA, USA) and TIRITC-labeled donkey anti rabbit IgG (1:200, A-16028, Invitrogen, Carlsbad, CA, USA) for overnight at 4 °C. After three rinses with PBST, the CNS were dehydrated using an ethanol series and cleared using methyl salicylate.

To count the PDF-ir varicosities, we sampled the CNS every 4 h from Day5 ZT12 to Day6 ZT 8 under LD and SD conditions. During the dark period the CNS was dissected under a red LED light (M-BL-E26-7W-660 nm, MS-system, Utsunomiya; approximately 390 lx). We simultaneously dissected five CNSs from SD larvae and five from LD larvae at the same time at ZT12, ZT16, ZT20, ZT24 (Day5), ZT4, and ZT8 (Day6). SD and LD CNSs were fixed with 4% PFA at RT for 4 h. The CNS was removed during the dark period and kept in the dark during fixation. After fixation, the CNSs were washed in 0.1 M PBST and kept at 4 °C until use. All samples (N = 60) collected from Day5 ZT12 to Day6 ZT 8 were processed at the same time for immunohistochemistry, from blocking before primary antibody incubation to the final step. The CNS was blocked in 5% NDS for 1 h at RT, and incubated in mouse anti-PDF antibody (1:100, RRID:AB_760350, PDF C7, DSHB, Iowa, USA) for 3 d at 4 °C. After primary antibody incubation, the CNS was washed in 0.1 M PBST and kept in the final wash at 4 °C overnight. After one wash at RT for 1 h, the CNS was blocked with 5% NDS for 1 h at RT and incubated with Alexa Fluor 488 labeled goat anti-mouse IgG (1:200, A11001, Thermo Fisher Scientific), in 5% NDS in PBST at 4 °C for 2 d. After three rinses with PBST, the CNS was dehydrated using an ethanol series and cleared using methyl salicylate.

### Backfills followed by PDF immunohistochemistry

The ring gland was severed one-third from the distal end with micro-scissors, and 20 mM neurobiotin (SP-1120, Vector Lab.) was introduced through the cut end using a sharpened pipette for a unilateral backfill. Using this labeling, contralateral pars lateralis (PL-c) neurons and ipsilateral PL (PL-i) neurons were labeled unilaterally. The pipette was made from borosilicate glass capillaries (GD-1.5; Narishige, Tokyo, Japan) using a flaming/brown micropipette puller (P-97; Sutter Instruments, Novato, California, USA). Backfilling was performed for 2–3 h at RT. After backfills, the CNS was fixed with 4% PFA overnight, rinsed 3 times with 0.1 M PBST, and incubated in mouse anti-PDF antibody (1:100, RRID:AB_760350, PDF C7, DSHB) for 3 d at 4 °C. The CNS was then incubated with Alexa Fluor 488 labeled goat anti-mouse IgG (1:200, A11001, Thermo Fisher Scientific) and an avidin–biotin complex (PK-4000, Vector Lab.) at 4 °C for 1 d followed by streptavidin Alexa fluor 647 (1: 200, S21374, Thermo Fisher Scientific) for overnight at 4 °C, and then processed for dehydrated with an ethanol series and cleared with methyl salicylate.

### Single-cell reverse transcription nested PCR

Single-cell reverse transcription nested PCR was performed as described by Hasebe and Shiga (2021a). We first labeled the PL-c cells of Day5 larvae under LD and SD conditions by backfills using Alexa Fluor 488 with dextran 3000 MW (D34682, Thermo Fisher Scientific) for 2–3 h at RT. PL-c cells were specified as cells stained in the brain hemisphere contralateral to the dye-filled side of the RG. Pipettes for cell collection were prepared from borosilicate glass capillaries (GD-1.5; Narishige, Tokyo, Japan) using the flaming/brown micropipette puller (P-97). Cells labeled with Alexa Fluor 488 were isolated using a pipette between ZT6 and ZT8 under an upright microscope (ECLIPSE FN1; Nikon, Tokyo, Japan) equipped with an ORCA-spark digital CMOS camera (C11440-36U; Hamamatsu Photonics, Shizuoka, Japan). A single collected cell was placed in a mixture of 4 μL of reverse transcriptase (FastGene Scriptase II cDNA Synthesis 5 × ReadyMix; NIPPON Genetics, Tokyo, Japan) and 16 μL of pure water, and reverse transcription PCR (RT-PCR) was performed to synthesize cDNA by TaKaRa PCR Thermal Cycler Dice (Takara Bio, Shiga, Japan). PCR mix solution was prepared with 1 μL of template cDNA, 12.5 μL KAPATaq Extra Hot Start ReadyMix with dye (Kapa Biosystems-Roche, Basel, Switzerland), 0.25 μL forward primers (20 μM), 0.25 μL reverse primers (20 μM) and 11 μL pure water, and then PCR was performed. The primary and secondary PCRs were performed by an initial heat denaturation at 95 °C for 3 min and 35 cycles of denaturation at 95 °C for 30 s, 48 °C for 30 s, and 72 °C for 40 s. One microliter of the reverse transcription reaction solution was used as template cDNA in the primary PCR. One microliter of the primary PCR solution was used as the template DNA in the secondary PCR. After the nested PCR, electrophoresis was performed on 1.5% agarose gel using submarine electrophoresis device MARINE23ST (FUJIFILM Wako Pure Chemical Corporation, Osaka, Japan). For electrophoresis, a 50 bp DNA ladder (NE-MWD50, Nippon Genetics) was used to measure the length of the PCR products. Agarose gels were incubated in Midori Green Advance solution (NE-MG04; Nippon Genetics) for at least 60 min. Using the Gel Documentation System AE-6932GXCF with a CCD camera Controller AE-6905CF (ATTO Corporation, Tokyo, Japan), we photographed the PCR product bands in the agarose gels. Target gene sequences were searched in the *S. similis* RNA-Seq database using tBLASTn (https://blast.ncbi.nlm.nih.gov/Blast.cgi?PROGRAM=tblastn&PAGE_TYPE=BlastSearch&LINK_LOC=blasthome) and ORF Finder (https://www.ncbi.nlm.nih.gov/orffinder/) using *D. melanogaster* amino acid sequences (Table 1). Primer3 (https://primer3.ut.ee/) and ApE-A plasmid Editor v2.0.61 (Davis and Jorgensen 2022) were used to create primer sets for primary and secondary reverse transcription nested PCR (Table 2).

**Table 1:** The contig list of *Sarcophaga similis*

Putative gene	Accession No	Length (bp)	ORF (aa)*	Identity to *D. melanogaster*	
				%	Acc. Number
*Ss_rpl32*	LC782570	1014	133	95.52	NP_733339.1
*Ss_ptth*	LC782571	395	34	58.06	NP_001303304.1
*Ss_pdf receptor*	LC782572	1395	376	59.48	NP_570007.2
*Ss_snpf receptor*	LC782573	2571	488	63.17	NP_001262086.1
*Ss_glycine receptor*	LC782574	1543	421	59.33	NP_524131.1
*Ss_glucl*	LC782575	1065	277	100	NP_001287409.1
*Ss_pdf*	LC815001	276	91	47.92	NP_524517.1
*Ss_snpf*	LC832170	556	148	48.93	NP_724239.1

**Table 2:** The primer list for reverse transcription nested PCR

gene	Usage	Forward (5′ → 3′)	Reverse (5′ → 3′)	Product size (bp)
*rpl32*	1st	ATGACCATTCGTCCAGCATATAGG	TGTGAACGAACACGACCATTG	392
	2nd	TGTTAAGAAGCGCACCAAGC	ACTTCTTGAAGCCAGTTGGGAG	194
*ptth*	1st	TGATTTGGTGGATTTGGGTCAGC	TCTTAAATCATCCGGTAGCCATGC	214
	2nd	TTCCACGGTACTTGCTTAATGC	ATTCCAATGGACGGCATACC	99
*pdf receptor*	1st	TGCAGGTGGTGATACGTTTAAG	CAATGGCAACAGAACGATGG	521
	2nd	GTGACAATCACGACCCTACC	GCGTGTCTGTTCAATATCACTGG	437
*snpf receptor*	1st	ATGTATTTCTCCACCACAGCAC	GAGTGAGCCACAAAGAAGAGTAAC	1004
	2nd	GGGTGTCTTTGGCAATGTATTGG	GCAGCCAAGACAGACCAAAC	806
*glycine receptor*	1st	AAACGCACAACAACAGCAAC	CAGTGCTGGTGGCAAATTC	665
	2nd	CAGCAATCACCACGCCATC	CTACGATTACCACCCGCCTG	581
*glucl*	1st	AGGAGGGCCATTTCCATAACATC	TCGAGCAGGGCACCGAATAC	586
	2nd	TGGTTCCGTGCTATACAGTATTCG	CGGTCCAGACATCAATAGCCTTC	491

### Electrophysiological analysis of neurotransmitter perfusion effects on PL-c neuronal activities

We recorded the electrophysiological activity of PL-c neurons. After fluorescently labeling the PL-c neurons with backfill, the larvae were dissected at ZT8. The whole brain was carefully moved into a handmade recording chamber (Hasebe & Shiga 2021b). The recording chamber was filled with a fly extracellular solution (ion components: 101 mM NaCl, 3.0 mM KCl, 1.0 mM CaCl_2_, 4.0 mM MgCl_2_・6 H_2_O, 5.0 mM glucose, 1.25 mM NaH_2_PO_4_・2 H_2_O, and 20.7 mM NaHCO_3_, pH was adjusted to approximately 7.2 with NaOH) (Flourakis et al. 2015). Recording pipettes were made from borosilicate glass capillaries (GD-1.5, Narishige) using the flaming/brown micropipette puller (P-97, Sutter Instruments). Recording pipettes were filled with a normal intracellular pipette solution (ion components:130 mM K^+^-gluconate, 4.0 mM NaCl, 1.0 mM MgCl_2_∙6 H_2_O, 0.5 mM CaCl_2_, 10 mM EGTA, and 10 mM HEPES, pH 7.2, adjusted with KOH) (Hasebe and Shiga 2022, 2021a), and tip resistance of recording pipettes was approximately 5–11 MΩ. PL-c neurons labeled with Dextran Alexa Fluor 488; 3,000 MW (D34682, Thermo Fisher Scientific) were detected under an upright microscope with a mercury lamp fluorescence irradiation device (ECLIPSE FN1, Nikon) and an ORCA-spark digital CMOS camera (C11440-36U, Hamamatsu Photonics). The recording pipette approached the fluorescently labeled PL-c cells. We formed a giga seal by applying negative pressure and then broke the cell membrane using a zap voltage pulse in the whole-cell patch clamp mode. We recorded the spontaneous firing of PL-c neurons in a current clamp mode. Electrophysiological recordings were performed using an Axopatch 200 B, Digidata 1550 B, and pCLAMP 11.0.3 software (Molecular Devices, Sunnyvale, CA, USA).

Perfusion of each neurotransmitter was performed using a Peristaltic Pump/MINIPULS 3 (M&S Instruments Inc., Osaka, Japan). After 6 min or more baseline recording, we perfused 1 µM PDF peptide NSELINSLLSLPKNMNDA-NH_2_ (APREST88038, MERCK, Darmstadt, Germany) or 1 µM short-neuropeptide F (sNPF1) AQRSPSLRLRF-NH_2_ (GenScript, Tokyo, Japan), the sequence of which corresponds to the predicted *S. similis* PDF or *S. similis* sNPF (Table 1), or 1 mM L-glutamic acid monosodium salt hydrate solution (L-glutamate, G1626, Sigma-Aldrich, St. Louis, MO, USA) for 2 min. The perfusion concentration for PDF was based on Gestrich et al. (2018). Subsequently, the normal fly extracellular solution was perfused for washout. We calculated the instantaneous frequency and number of firing at 0.5 min to 2.0 min before the neurotransmitter perfusion as “Before.” The instantaneous frequency and number of firing at 0.5 min to 2.0 min after the PDF, sNPF1 and L-glutamate perfusion were calculated as “PDF”, “sNPF1” and “L-Glutamate,” respectively, and those at 8.5 min to 10 min after the start of wash-out were calculated as “Wash-out.” Electrophysiological data were analyzed using the Clampfit software version 10.7 (Molecular Devices, Sunnyvale, CA, USA).

### Microscopy

Fluorescent images were acquired using a confocal laser scanning microscope (LSM 710; Carl Zeiss, Oberkochen, Germany) equipped with an objective lens (EC Plan-Neofluar 20 × /0.50 M27 and Plan-Apochromat 63 × /1.4 oil DIC M27, Carl Zeiss). Alexa Fluor 488 and TRITC were excited using an argon laser (488 nm) and Alexa Fluor 647 was excited using a red HeNe laser (633 nm). The emission wavelengths were set at 523 nm for Alexa 488, 562 nm for TRITC, and 697 nm for Alexa 647. Optical sections were reconstructed using an image processing software (Zen 3.5, Carl Zeiss). For three-dimensional reconstruction, laser scanning microscopy images were processed using image-processing software (Amira 2019, Thermo Fisher Scientific). Double labeled processes of the two types of neurons were manually segmented. The generated surface and surface views were used for the 3D reconstruction.

### Counting of PDF-immunoreactive varicosities

We examined the number of varicosities using the multi-point tool in ImageJ software (Wayne Rasband, National Institute of Health, Bethesda, MD, USA). First, the labeled samples were named randomly such that the inspector was blinded to the ZT and photoperiodic conditions of the sample. The inspector counted the varicosities in each slice of the sample. To avoid double counting of the same varicosity, each counted varicosity was labeled with a dot with a number. The same investigator conducted blind counting twice for each sample. If there was a difference in the number between the first and second counts of more than 10% of the first count, the third count was performed. When the difference between the third and first counts was less than 10% of the first count, or the difference between the third and second was less than 10% of the second count, we stopped counting. The average number of the two counts with 10% or less difference was calculated. With this method, there was no fourth count.

### Statistical analysis

For comparison of diapause incidences, we performed Tukey’s multiple comparisons for proportions using Excel TOUKEI ver.7 (ESUMI Co., Ltd., Tokyo, Japan). For electrophysiological data analyses, we used Kyplot 6 software (KyensLab, Tokyo, Japan). We first checked whether the data was normally distributed by Shapiro–Wilk test. Then, for comparisons between multiple groups where the data are normally distributed, Tukey test was performed. For data comparisons that were not normally distributed, Steel–Dwass test was performed. For Comparison of varicosity numbers, we performed two-way ANOVA with Student’s *t*-test with Holm correction by Excel TOUKEI ver.7. *P* < 0.05 was set as statistically significant.

## Results

### Photoperiodic response of *S. similis* larvae

**Fig. 1:**
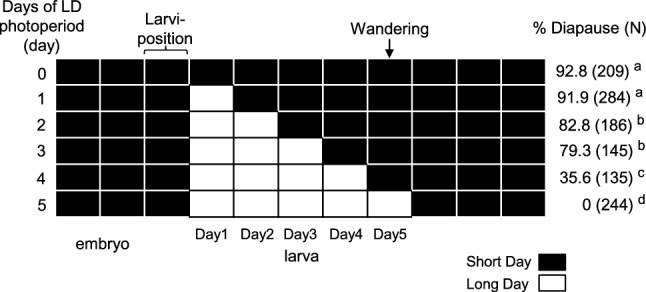
Larval photoperiodic response in *Sarcophaga similis*. Rectangular arrays indicate photoperiodic schedules. Larvae were subjected to either of six photoperiodic schedules (receiving 0–5 long days (LD) against short days). Diapause incidences and individual numbers are on the right edge. Different letters indicate significant differences in diapause incidences (*P* < 0.01, Tukey’s multiple comparisons for proportions)

First, we examined the photoperiodic sensitivity of the fly strains. SD larvae were divided into six groups, and each group was exposed for 0–5 d after larviposition to LD conditions (Fig. 1). When *S. similis* larvae were reared totally under SD conditions, 92.8% (N = 209) entered pupal diapause. However, the diapause incidence was decreased significantly when 2 d or more LD were given. When larvae were exposed to LD conditions for 4 d, the diapause incidence became 35.6% (N = 135). LD exposure for 5 d completely averted diapause (Fig. 1).

### Internal structure of the ring gland

**Fig. 2:**
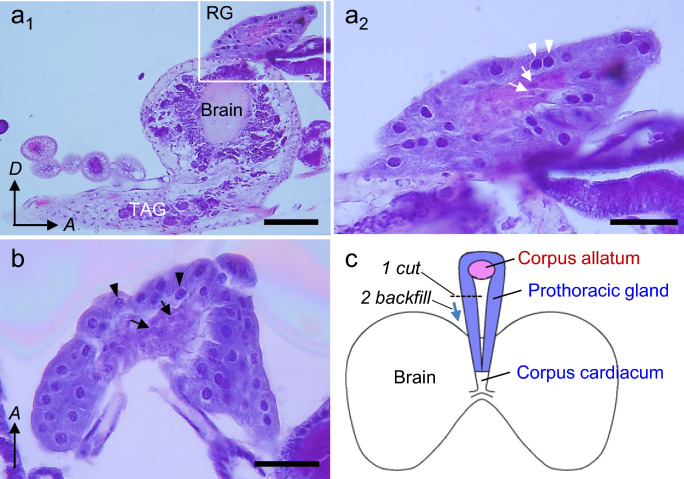
Hematoxylin–eosin staining of the brain and ring gland of *Sarcophaga similis* larvae (Day5 under long-day conditions)*.* **a** Sagittal section. A rectangle in **a**_**1**_ is enlarged in **a**_**2**_. A part of the thoracico-abdominal ganglia (TAG) is also found. **a**_**2**_, **b** The distal part of the ring gland (RG). The eosin-stained cells (arrows) are surrounded by several hematoxylin-stained cells (arrowheads). **c** A schematic illustration of the RG and brain. The corpus allatum and prothoracic gland are shown in different colors. The RG was cut and dye was filled to the brain to stain neurons innervating the prothoracic gland and corpus allatum. *A*, anterior; *D*, dorsal. Sales: 100 µm in **a**_**1**_, 50 µm in **a**_**2**_ and **b**

To characterize the neurons innervating the PG region, we histologically examined the ring gland (RG) in paraffin sections. Two cell types have been identified in the middle and distal regions of the RG. The region close to the distal end of the RG was stained in weak magenta color (Fig. 2a). Cell size in the magenta-colored distal region was ca. 15 μm. Cells outside of these cells were larger and colored in dark blue, and their diameter was ca. 25 μm (Fig. 2b). Following Enya et al. (2014), we identified cells in weak magenta color the corpus allatum cells and those outside the corpus allatum region PG cells (Fig. 2c).

### Morphological connections between PDF-LNs and PL-c neurons

**Fig. 3:**
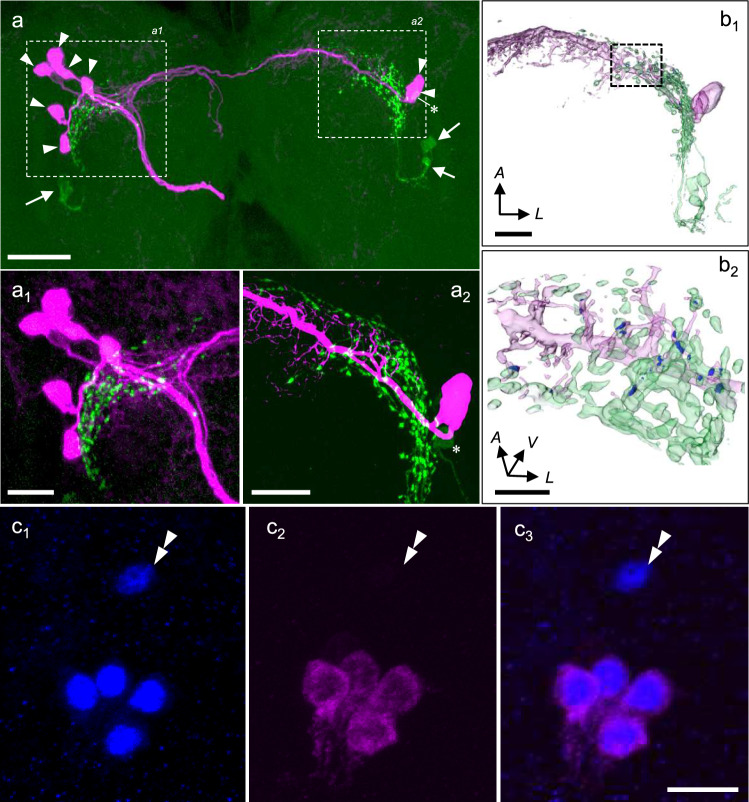
Neuroanatomy of pigment-dispersing factor (PDF), PERIOD (PER) immunoreactive neurons and neurons projecting to the prothoracic gland (PG) region in *Sarcophaga similis* larvae (Day5 under long-day conditions). Dorsal view, upper to the anterior. **a** Double labeling of backfills from the PG region (magenta) and PDF immunohistochemistry (green). Unilateral backfills labeled six ipsilateral pars lateralis (PL-i) cells and two contralateral pars lateralis (PL-c) cells (magenta, arrowheads). A magnified view of PL-i neurons (**a**_**1**_) and PL-c neurons (**a**_**2**_) is shown. PDF-immunoreactive fibers are found at the dorsal protocerebrum protruding from cells (arrows), and several immunoreactive varicosities are found along the terminal arborizations close to the PL neurons. **b** A three-dimensional reconstruction of the confocal image for the PDF-immunoreactive fibers (green) and PL-c fibers (magenta). **b**_**2**_ is a magnified view of the dotted line in **b**_**1**_. Overlap of the PDF-ir and PL-c fibers is indicated in blue. **c** Double immunohistochemistry using antibodies against PER (blue, **c**_**1**_) and PDF (magenta, **c**_**2**_) in the brain lateral region. Four among five PER immunoreactive cells were also labeled with PDF (**c**_**3**_), One cell (double arrowheads) was only PER immunopositive. **a**, **a**_**1**_, A stack of 97 confocal sections with a pixel size of 0.52 μm and voxel-depth 1.0 μm; **a**_**2**_, a stack of 81 confocal sections with a pixel size of 0.13 μm and voxel-depth 0.3 μm; **c**, a stack of 69 confocal sections with a pixel size of 0.11 μm and voxel-depth 0.4 μm. Asterisk, One PDF-immunoreactive cell found close to the PL-c. *A*, anterior; *L*, lateral; *V*, ventral. Sales: 50 µm in **a;** 20 µm in **a**_**1**_, **a**_**2**_ and **b**_**1**_**;** 10 µm in **b**_**2**_ and **c**_**3**_

To examine the anatomical relationship between the neurons projecting to the PG and PDF-ir neurons, we performed PDF immunohistochemistry after filling neurobiotin from approximately one-third of the distal end of the RG to the brain for labelling the neurons projecting to the PG and corpus allatum regions (Fig. 2c). Unilateral backfill from the RG revealed two cell clusters in the PL (Fig. 3a): Six cells in the ipsilateral PL (PL-i, Fig. 3a_1_) and two cells in the contralateral PL (PL-c, Fig. 3a_2_). PL-c neurons bore fine branches along the axon protruding from the cells (Fig. 3a_2_). Four PDF-ir lateral neurons were found in each brain hemisphere that extended dorsomedially to the protocerebrum, as reported previously (Fig. 3a, Hirata and Shiga 2023). PDF-ir varicosities appeared near the fine branches of the PL-i and PL-c neurites Fig. 3a_1_, a_2_). The PDF-ir and PL-c fibers were three-dimensionally reconstructed (Fig. 3b_1_, b_2_). Several PDF-ir varicosities were found to overlap with the fibers of PL-c neurons (blue puncta in Fig. 3b_2_).

Double immunohistochemistry using PER and PDF antibodies showed that all four PDF-ir cells were labeled with the PER antibody, while one cell was only PER-immunopositive (Fig. 3c). Colocalization was confirmed in three brain samples. These results suggest that all PDF-ir neurons contain PER and send fibers very close to the neurites of PL-c neurons projecting to the PG region.

### PL-c neurons expressing *ptth*, *glucl, *and partly *snpfr*

**Fig. 4:**
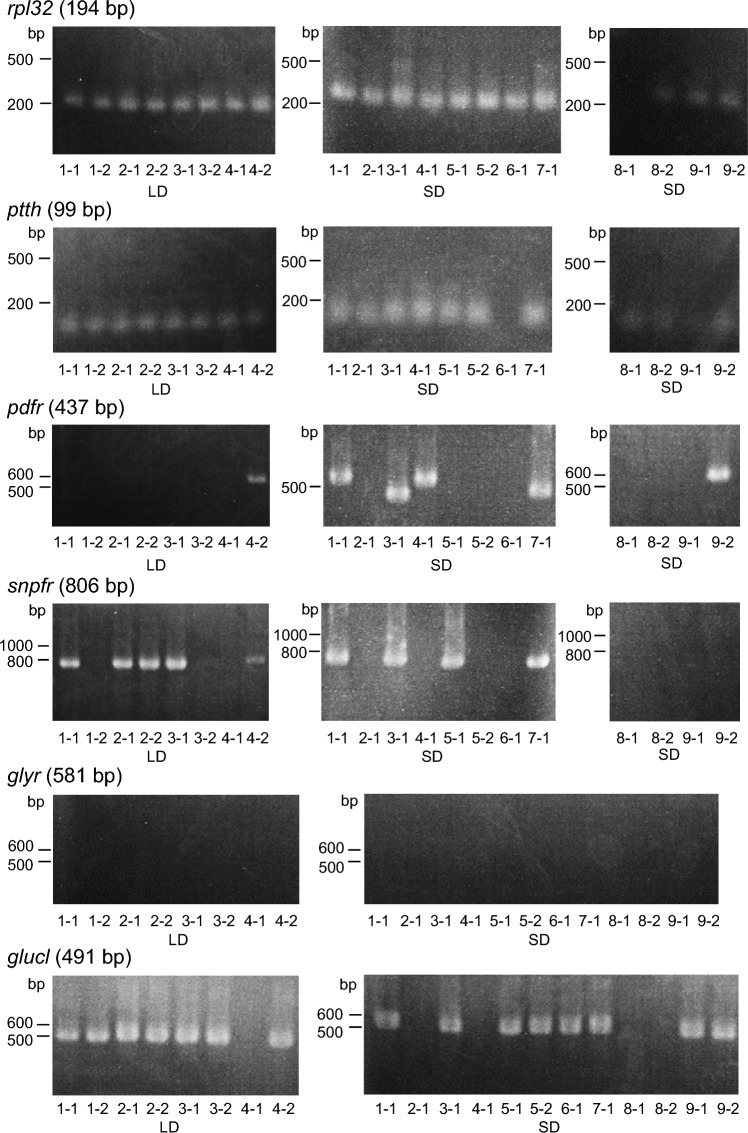
Single cell reverse transcription nested PCR of PL-c neurons of *Sarcophaga similis* larvae. Images showing expression of *ribosomal protein L32* (*rpl32*; control gene), *prothoracicotropic hormone* (*ptth*), *pigment-dispersing factor receptor* (*pdfr*), *short-neuropeptide F receptor* (*snpfr*), *glycine receptor* (*glyr*), *glutamate-gated chloride channel* (*glucl*) in 20 PL-c cells from 13 Day5 larvae. The second-transcript length in the reverse transcription nested PCR is shown after gene name. The number below the photograph shows individual-cell codes. LD, long days; SD, short days

In the *D. melanogaster* larval brain, two pairs of neurons contralaterally projecting to the PG express *ptth* (McBrayer et al. 2007). Thus, we examined expression of genes, including *ptth* in the two pairs of PL-c neurons in *S. similis*. We collected one or two PL-c cells per brain from 13 larvae (a total of 20 cells, 8 cells from LD and 12 cells from SD larvae) and performed single-cell RT-PCR*.* Nineteen of the 20 clearly expressed *ribosomal protein L32* (*rpl32*) used as a positive control (95% in total). *ptth* was expressed in all 8 cells from LD larvae and in 10 of 12 cells from SD larvae (Fig. 4, 90% in total). These results indicated that the two PL-c neurons were *ptth*-expressing neurons.

PDF, sNPF, and glycine (Gly) are neurotransmitters released from s-LNvs, and glutamate (Glu) is released from DN1s in *D. melanogaster* (King and Sehgal 2020). Next, we examined whether these neurotransmitters could signal PL-c neurons by studying the expression of their receptors. In the nested PCR, *pdf receptor* (*pdfr*) transcripts with the target second PCR product size (437 bp) were found in two PL-c cells from SD larvae and in none from LD larvae (10% in total). The first PCR product (521 bp) without the second one was found in some cells, suggesting non-specific binding of the products due to unexpected primer-binding sites. We considered these as negative expressions. Transcripts of *short-neuropeptide F receptor* (*snpfr*) were detected in five out of eight PL-c cells from LD larvae and in four out of 12 cells from SD larvae (45% in total). No cells expressed *glycine receptor* (*glyr*). An inhibitory glutamate receptor, *glutamate-gated chloride channels* (*glucl*) was expressed in seven out of 8 PL-c cells from LD larvae and in eight out of 12 cells from SD larvae (Fig. 4, 75% in total). These suggest that *ptth*-expressing PL-c neurons mainly express *glucl,* whereas some neurons may express *snpfr.* Receptor expression did not differ between SD and LD larvae.

### L-glutamate and sNPF1, but not PDF peptide, acutely and strongly suppress spontaneous firing activities of PL-c neurons

**Fig. 5:**
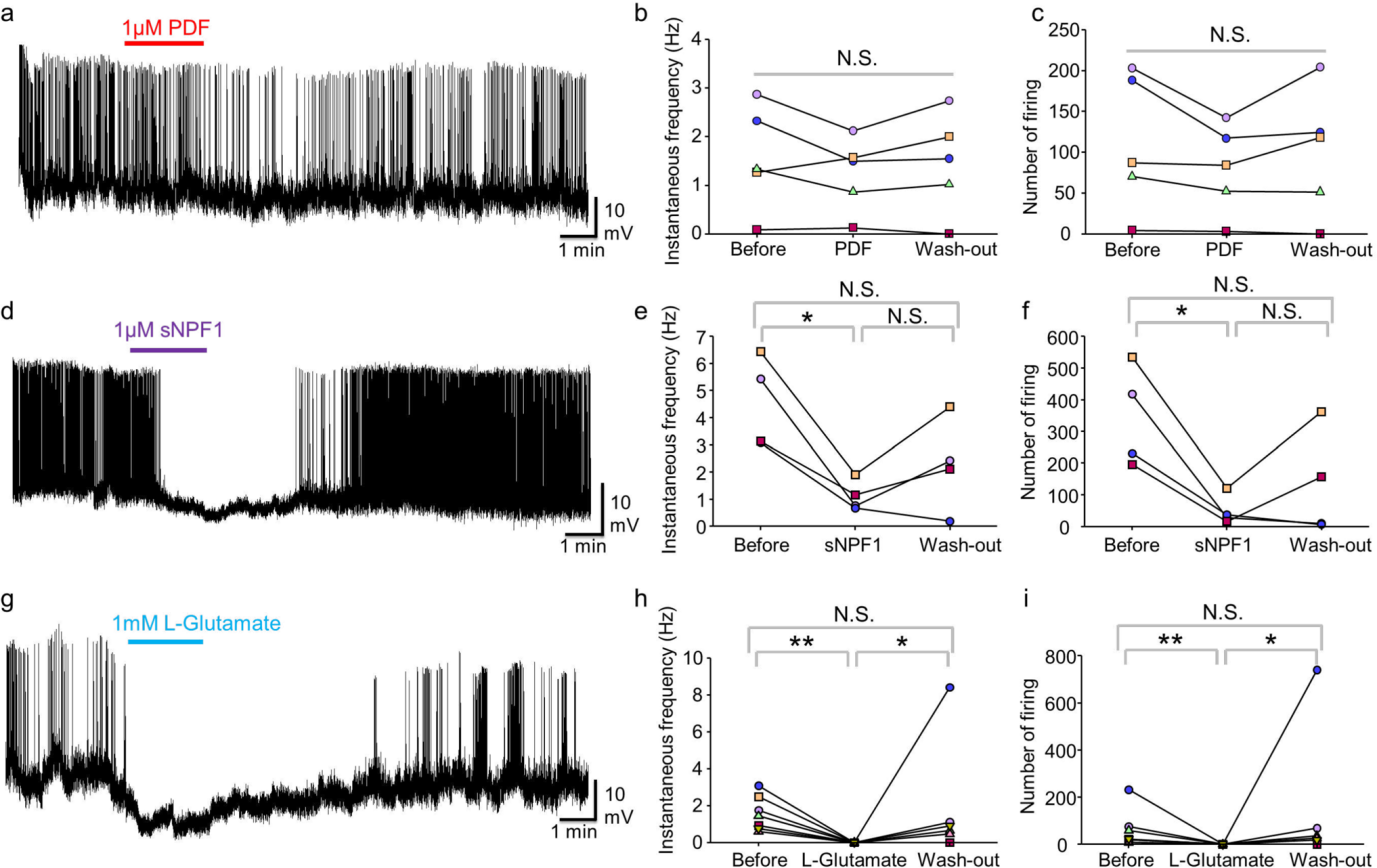
Perfusion effects of PDF, sNPF and L-glutamate on spontaneous firing activities of PL-c neurons of *Sarcophaga similis* larvae (Day5 under long-day conditions). **a**, **d, g** Representative traces showing effects of 1 µM of PDF perfusion (**a**), 1 µM of sNPF1 perfusion (**d**), and 1 mM of L-glutamate perfusion (**g**) on the spontaneous firing activity of PL-c neurons. (**b**, **c**, **e**, **f**, **h**, **i**) Line graphs showing the instantaneous frequency (**b**, **e**, **h**) and number of firing events (**c**, **f, i**) in 1.5 min before perfusion (Before), during perfusion (PDF, sNPF1or L-Glutamate), and after perfusion (Wash-out) within each PL-c neuron (**b**, **c**: *n* = 5; **e**, **f**: *n* = 4; **h**, **i**: *n* = 7). (**b**, **c**, **e**, **f**) Tukey test, (**h**, **i**) Steel–Dwass test, *: *P* < 0.05, **: *P* < 0.01, N.S.: not significant

As 75% of PL-c neurons expressed the inhibitory Glu receptor *glucl* and 45% expressed *snpfr* (Fig. 4), we analyzed the effects of L-glutamate and sNPF1 on PL-c neuron activity using electrorheological perfusion analyses. We also examined PDF effects on PL-c neurons, because PDF-ir varicose fibers were found close to PL-c fibers (Fig. 3a_2_). PL-c neurons in Day5 LD larva exhibited spontaneous firing activity (Fig. 5a, d, g); Perfusion with the PDF peptide had no significant effect on neither instantaneous frequencies nor firing numbers of the spontaneous activity (Fig. 5a, b, c, *P* > 0.05, Tukey test). In contrast, sNPF1 perfusion significantly and acutely inhibited the spontaneous firing activity in PL-c neurons (Fig. 5d, e, f,  *P* < 0.05, Tukey test). L-glutamate perfusion completely abolished the spontaneous PL-c firing activity, which recovered after wash-out (Fig. 5g, h, i,  *P* < 0.05, Steel–Dwass test). These results and single-cell PCR results suggest that Glu and sNPF act as inhibitory signals for *ptth* expressing PL-c neurons.

### Comparison of PDF-ir varicosities in the dorso-lateral protocerebrum between LD and SD conditions

**Fig. 6:**
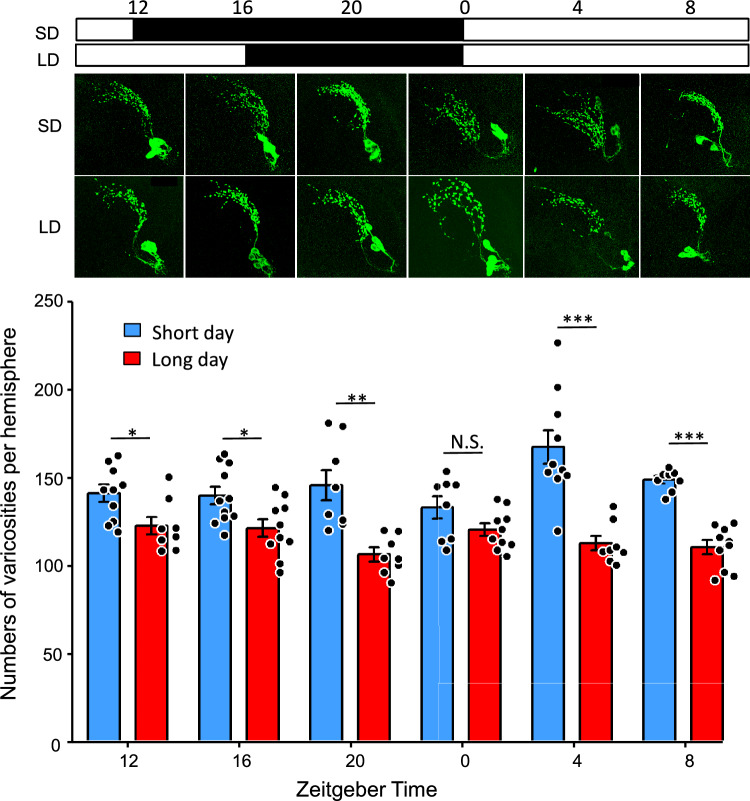
Comparison of varicosity numbers of pigment-dispersing factor (PDF) immunoreactive fibers between long-day and short-day conditions in *Sarcopharga similis* larvae. Representative images showing PDF immunoreactive neurons every four Zeitgeber times. The white and black bars above represent light and dark periods, respectively. Dots in the graph indicate numerical values of each hemisphere, and columns and bars indicate mean ± standard error. Larvae, N = 5; cells, n = 8 – 11. After two-way ANOVA (see Table 3), Student’s *t*-test with Holm correction was applied (*: *P* < 0.05, **: *P* < 0.01, ***: *P* < 0.001, N.S.: not significant)

To determine whether any photoperiodic differences were detected in PDF- and PER-ir LNs, we compared the number of PDF-ir varicosities in the dorsal protocerebrum per hemisphere between LD and SD larvae at six timepoints on Day5–6 (Fig. 6). No significant effects of ZT on varicosity numbers per hemisphere were found (Table 3, two-way ANOVA, *P* = 0.12), but a significant difference was found by photoperiod (Table 3, two-way ANOVA, *P* = 2.79 × 10^–15^,). Because an interaction effect was detected between photoperiod and ZT (two-way ANOVA, *P* = 1.69 × 10^–3^), we compared the varicosity number between photoperiodic conditions at each ZT. The varicosity numbers in SD larvae were significantly higher than those in LD larvae, except at ZT0 (Student’s *t*-test with Holm correction, *P* < 0.05).

**Table 3:** Two-way ANOVA assessing effects of photoperiod and Zeitgeber in varicosity numbers of pigment-dispersing factor immunoreactive neurons in *Sarcophaga similis*

		Df	Sum Sq	Mean Sq	F	P
Photoperiod		1	24,836	24,836	88.16	2.79E-15 ***
Zeitgeber		5	2549	510	1.81	0.11803
Photoperiod: Zeitgeber		5	5916	1183	4.2	0.00169 **
Residuals		97	27,327	282		

^**^P < 0.01; ***P < 0.001

## Discussion

### Morphological identification of PTTH neurons and PDF-LNs in *S. similis* larvae

In the RG of *D. melanogaster* and *P. terraenovae* larvae, the corpus allatum is located at the distal end and is surrounded by PG cells (Meurant and Sernia 1993; Siegmund and Korge 2001; Hamanaka et al. 2009; Enya et al. 2014). In *S. similis* similar histological distinction between the corpus allatum and surrounding cells was observed, and the surrounding cells must be PG cells. In the current unilateral backfill from the PG region, two PL-c neurons and six PL-i neurons were labeled. These projection patterns are consistent with those observed in *S. crassipalpis* (Giebultowicz and Denlinger 1985). Furthermore, *ptth* expression was confirmed in PL-c cells of *S. similis*. In *D. melanogaster* larvae, two PTTH neurons, named LP-PGs, per brain hemisphere contralaterally innervate the PG (Siegmund and Korge 2001; McBrayer et al. 2007). This strongly suggests that the two pairs of *ptth*-expressing PL-c neurons projecting contralaterally to the RG are PTTH neurons in *S. similis* larvae. In the tobacco hornworm *Manduca sexta*, two PTTH neurons per hemisphere project contralaterally (Agui et al. 1980; O'Brien et al. 1988). Thus, the number and projection patterns appeared to be common between moths and flies.

Using immunohistochemistry, five PER-positive LNs were found, as in a previous report (Yamamoto et al. 2017), and the current study showed that four of them were PDF immunoreactive. In *D. melanogaster* four pairs of small PDF neurons (called sPDFMe) in larvae develop as *per*-expressing s-LNvs of the adult brain (Helfrich-Förster 1995, 1997). One PDF-negative 5th LN develops to the adult 5th LN which shares fiber distribution patterns with the dorsal LNs rather than the s-LNvs (Helfrich-Förster et al. 2007; Schubert et al. 2018). In the present study, we characterized four PDF- and PER-positive LNs as PDF-LNs, and one PDF-negative PER cell as 5th LN in *S. similis* larvae.

In this study, we further found in *S. similis* that varicose fibers derived from PDF-LNs projected close to the arborization areas of PL-c (PTTH) neurons in the dorsal protocerebrum. Using a three-dimensional reconstruction of confocal images, we found that PDF-ir varicose fibers and fibers of PL-c (PTTH) neurons had morphological contact in the protocerebrum (Fig. 3b). In *D. melanogaster* larvae dorsally ascending fibers from the PDF neurons form an elongated, slightly curved field that follows the path of the axons of the PTTH neurons (Siegmund and Korge 2001; McBrayer et al. 2007). Furthermore, it has been shown by green fluorescent protein reconstitution across synaptic partners (GRASP) that PDF neurons in *D. melanogaster* larvae form synaptic connections with PTTH neurons, although their transmitters during the larval stage have not been identified (Gong et al. 2010; Yamanaka et al. 2013). These suggest that a direct signal transmission from PDF-LNs to PTTH neurons occurs also in *S. similis*.

### Possible neural circuitries including PDF-LNs and PTTH neurons

We then searched for transmitter candidates from PDF-LNs to PTTH neurons. In *D. melanogaster* larvae four LNs contain PDF (Kaneko et al. 1997) with two LNs co-expressing sNPF (Johard et al. 2009). Gly is secreted from adult s-LNvs (Frenkel et al. 2017), although it has not yet been carefully examined in larval clock cells. Then we first chose PDF, sNPF and Gly as potential PDF-LN transmitters to PTTH neurons in *S. similis* and examined their receptor expression in PTTH cells. No PL-c (PTTH) cells expressed *glyr*. But *snpfr* and *pdfr* expression were observed in 45 and 10% of PL-c cells, respectively. There could be two possibilities: the first one is that their expression is detected from adjacent cells which had been picked up together with a PL-c cell at the cell collection step, and the second one is that they are expressed in the PL-c cell but its expression level is too low to be detected in several cells. From pharmacological experiments, we think that the first possibility is more probable as for PDFR; the second one is for sNPFR. In *D. melanogaster* pharate adults, PTTH neurons respond to sNPF but not to PDF (Selcho et al. 2017). Recently the PTTH neurons in *D. melanogaster* were shown to express the sNPF receptor but not the PDF receptor (Cavieres-Lepe et al. 2024). Our results on *S. similis* support this. We need to locate *snpf*-expressing cells in *S. similis*.

We also examined Glu as a possible transmitter affecting PTTH neurons. In *D. melanogaster* larvae, Glu is not present as a transmitter in LNs but is in DN1 (Hamasaka et al. 2007; Daniels et al. 2008). Interestingly, in *S. similis* larvae the majority of PTTH cells expressed *glucl* and spontaneous activity of PTTH neurons was acutely inhibited by Glu. These results indicate that Glu directly inhibits PTTH neurons in *S. similis* larvae. Some PTTH neurons did not show *glucl* expression, suggesting its expression may not be constant. If Glu is produced in DNs as in *D. melanogaster*, DNs might directly suppress PTTH neurons. Also, there is a possibility that Glu is produced in PDF-LNs in case of *S. similis*.

It is known in *D. melanogaster* larvae that PTTH neurons are synaptically connected with *pdf-*expressing LNs to regulate light avoidance behavior in addition to controlling eclosion timing (Gong et al. 2010; Yamanaka et al. 2013). The light avoidance behavior appears from the first to the mid-third instar larvae, and then it disappears when wandering larvae emerge from dark food areas for pupariation (Sawin-McCormack et al. 1995). PTTH is considered to affect signaling components downstream of photoreceptors, such as the Bolwig’s organ (BO) for the light avoidance behavior (Yamanaka et al. 2013). In the neural circuits for light avoidance behavior, involvement of LNs and DNs is also shown, although LNs are dispensable (Mazzoni et al. 2005; Keene et al. 2011; Collin et al. 2012). DN1s of the larval brain contain Glu to suppress LNs via GluCl and this Glu signaling is suggested to suppress light avoidance behavior (Hamasaka et al. 2007; Collin et al. 2012).

From the anatomical and functional analysis of *D. melangaster* larvae, DN, LNs and PTTH neurons form neural circuits and probably serve the decision when (developmental timing) and where (light avoidance response) animals ecdyse (Yamanaka et al. 2013). We found suppression of PTTH neurons by Glu via GluCl and sNPF, and morphological contacts between PDF-LNs and PTTH neurons. Although we have no idea about the identity of the Glu-releasing neurons in *S. similis* larva yet, Glu and sNPF potentially from LNs or DNs may inhibit PTTH neurons to control light avoidance. Concomitantly the circuits including PDF-LNs, possibly DNs, and PTTH neurons may control developmental timing to produce diapause and nondiapause pupae depending on photoperiod. We still need to identify glutamatergic cells and sNPF cells connecting to PTTH neurons in *S. similis* larvae.

### Photoperiodic plasticity in PDF-ir fibers of PDF-LNs

The s-LNvs in *D. melanogaster* adults exhibit structural plasticity, in which the complexity of the axonal arbor is higher in the morning than at night, according to two different methods (Fernández et al. 2008; Petsakou et al. 2015). Daily structural plasticity of the s-LNv termini is not required for circadian timekeeping, but is required for full entrainment to environmental temperature fluctuations, and daily changes in the s-LNv termini may alter the sensitivity of the clock network to sensory signals (Fernández et al. 2020).

In *D. melanogaster* larvae, morphological plasticity of PDF neurons has been reported. The total dendrite length of PDF neurons does not show daily fluctuations, but exhibits photoperiodic differences (Yuan et al. 2011). It becomes longer and the response of PDF neurons to light via the photoreceptor BO is stronger under constant dark and SD conditions than under LD and constant light conditions (Yuan et al. 2011). Yuan et al. (2011) suggested that light exposure modifies dendritic areas to change the light sensitivity of PDF neurons through the BO, and this could facilitate adaptation to seasonal changes. Here, we also observed photoperiodic changes in PDF-LNs in terms of PDF-ir varicosity numbers in *S. similis* in which seasonal control of pupal diapause is apparent. Of the PDF-LNs, dendritic fibers in *D. melanogaster* (Yuan et al. 2011) and PDF-ir terminal varicosities in *S. similis* (the present study) commonly exhibited morphological increase under SD conditions. In *S. smilis* larvae, BO neuron terminals are located close to the PDF-LN dendritic regions (Hirata and Shiga 2023). BO neurons may carry photoperiodic information to modify the PDF content in PDF-LN terminals to affect indirectly the PTTH-endocrine cascade for the photoperiodic control of pupal diapause. Yamamoto et al. (2017) showed photoperiodic difference in PER immunoreactivity in LNs of *S. similis*, and this may relate to PDF-LN terminal varicosity change. The photoperiodic plasticity of PDF-ir neurons has also been reported in the cockroach *Leucophaea maderae*, in which LD conditions increased the number of PDF-ir cells in the optic lobe and lengthened their fibers (Wei and Stengl 2011). This suggests that functional clock neurons must have the capacity for photoperiodic modification while maintaining circuit stability of the circadian clock system.

Neural varicosities represent sites of synaptic release of transmitters (synaptic transmission) as well as non-synaptic release of peptides (volume transmission) (Agnati et al. 1995). In *D. melanogaster*, electron microscopy and expansion microscopy revealed that varicosities of s-LNv termini in the dorsal protocerebrum contain large dense-core vesicles for volume transmission, numerous small clear vesicles for synaptic transmission, and postsynaptic structures, indicating that the dorsal termini serve as both input and output sites (Yasuyama and Meinertzhagen 2010; Shafer et al. 2022). PDF release from sLNvs is independent of presynaptic active zone, strongly suggesting that PDF is released in volume transmission (Hofbauer et al. 2024). Different PDF-ir varicosity numbers in *S. similis* suggest two possibilities: a difference in PDF content without structural change of PDF-LNs or a difference in PDF-LN fiber structure. Although we cannot distinguish between these two, the differences in PDF-ir varicosity numbers suggest some change in connectivity strength through the PDF-ir varicosities between the PDF-LNs and their post- or presynaptic neurons in *S. similis*. There is a possibility that PDF-ir varicosities contain other transmitters, such as sNPF and Glu, that convey direct signals to PTTH neurons differently between the SD and LD conditions.

Increasing the release or input sites at varicosities strengthens the neural connections to post- or presynaptic neurons in the neuronal circuitry. Considering importance of clock neurons and PTTH neurons for the photoperiodic response controlling pupal diapause, photoperiodic plasticity in PDF-ir varicosity close to PTTH fibers may be involved in the photoperiodic mechanism in *S. similis* larvae. In the near future, the functional significance of the clock neuron-PTTH neuron circuitry in the photoperiodic control of pupal diapause should be clarified.

## Data Availability

We open sequence data in the public service, the statistical test result is in the Table, all other data will be open on request.
